# Supplementary material for: A BAFF/APRIL axis regulates obesogenic diet-driven weight gain
Source: Nat Commun. 2021 May 18;12:2911. doi: 10.1038/s41467-021-23084-1 (PMC8131685; doi:10.1038/s41467-021-23084-1)
Supplement: Supplementary file 1 — Supplementary Information [file 41467_2021_23084_MOESM1_ESM.pdf]

Supplementary Information

A BAFF/APRIL axis regulates obesogenic-diet driven weight gain

Chan et. al

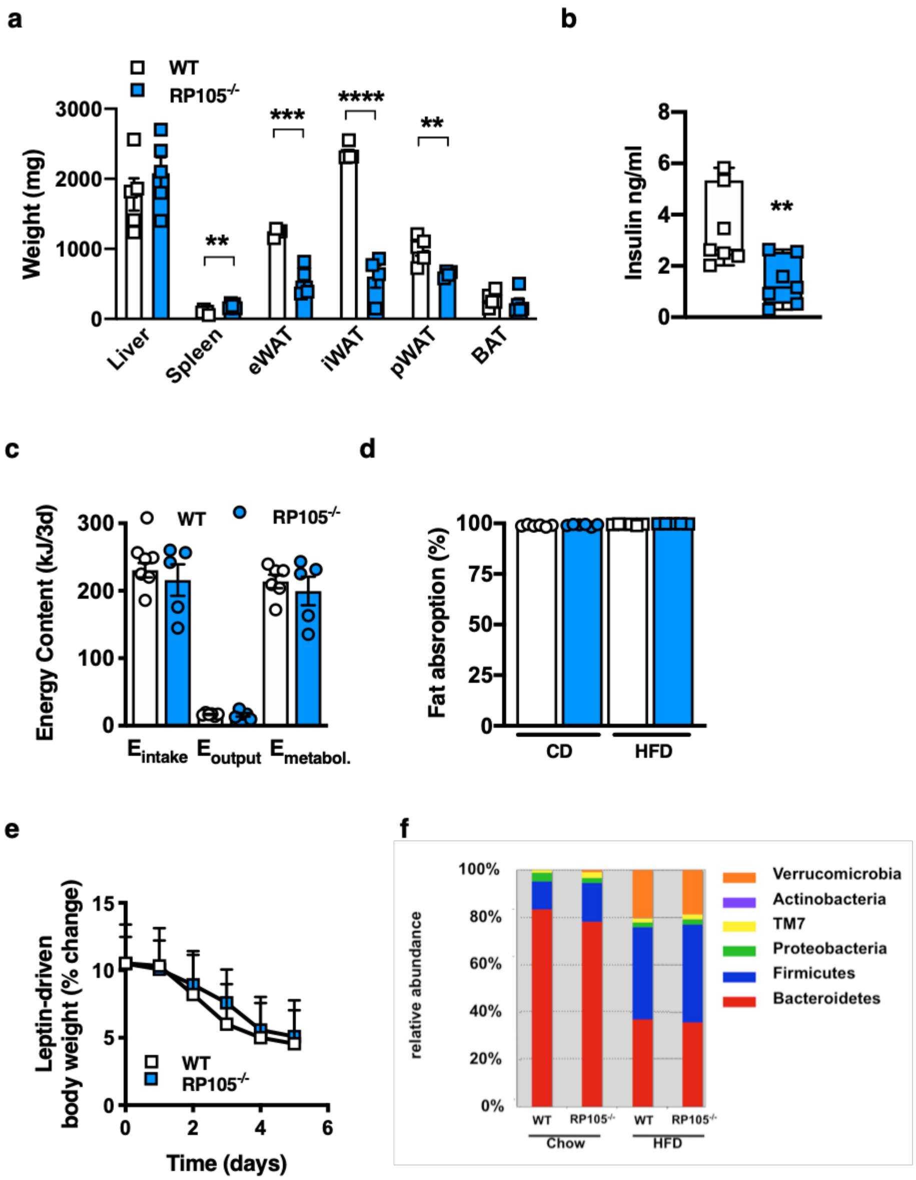

Supplementary Figure 1. Intrinsic lack of RP105 expression does not explain effects on protection from obesity pathogenesis. RP105$^{-/-}$ or WT mice were fed a low-fat chow diet (CD) or high-fat diet (HFD) for 24 weeks. (**a**) Various tissue weights as indicated at time of harvest. (**b**) Fasting serum insulin levels at time of harvest, quantified by colorimetric assay. (**c**) Nutrient and (**d**) fat absorption at 16 weeks on HFD. (**e**) Body weight after 4 doses of leptin or vehicle control administration at 6 weeks on HFD. (**f**) Phylum composition of intestinal microbiota in WT and RP105$^{-/-}$ placed on chow or HFD. (**a-f**) Representative of 3 independent experiments, n = 6-8/condition. (**a, c-e**) For bar and line graphs, data represents mean +/- SEM. (**b**) For box plots, the midline represents the mean, boxes represent the interquartile range and whiskers show the full range of values. (**a-e**) Unpaired two-tailed Student's t-test. (a-b) **$p < 0.01$, ***$p < 0.001$, ****$p < 0.0001$. Source data are provided as a Source data file.

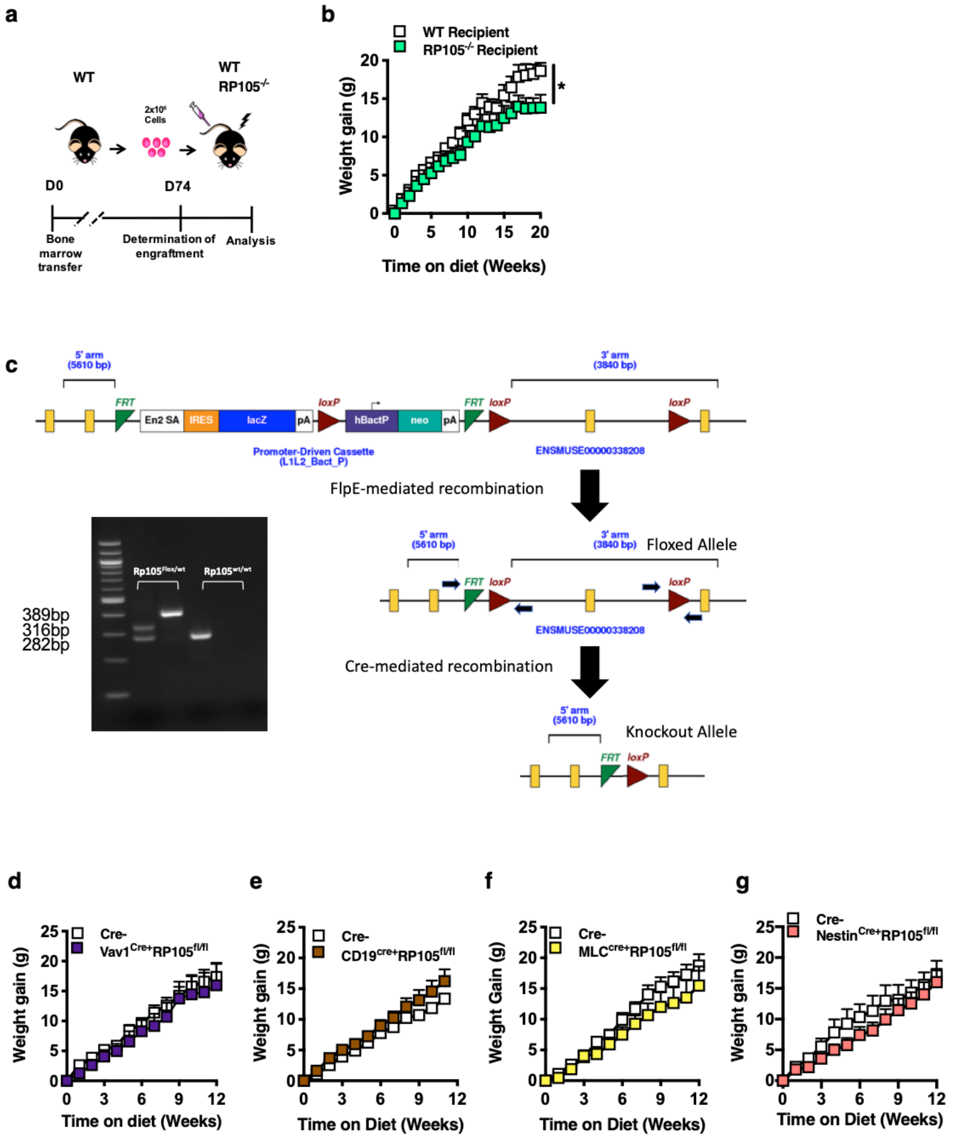

Supplementary Figure 2. Utilization of a conditional RP105 deletion in multiple cell types does not reveal the protective locus of effect from obesity development. (**a**) Scheme of reciprocal bone marrow transfer. (**b**) Weight gain of $RP105^{-/-}$ and $WT^{-/-}$ recipients fed HFD for 20 weeks. (**c**) Scheme of RP105 flox mouse design. Expected WT allele size 282bp and expected RP105 flox allele size 316bp. (**d-g**) RP105 conditional knockout mice fed HFD for 12 weeks. (**d**) Deletion in total immune cells ($Vav1^{cre}$). (**e**) Deletion in B cells ($CD19^{cre}$). (**f**) Deletion in skeletal muscle (MLC^{cre}). (**g**) Deletion in central and peripheral nervous system ($Nestin^{cre}$). (**a-b**) Representative of 2 independent experiments, n = 6/condition. (**d-g**) Representative of 2 independent experiments, n = 4-8/condition. (**b, d-g**) For line graphs data represents mean +/- SEM. (**b, d-g**) Area under the curve. (**b**) $*p < 0.05$. Source data are provided as a Source data file.

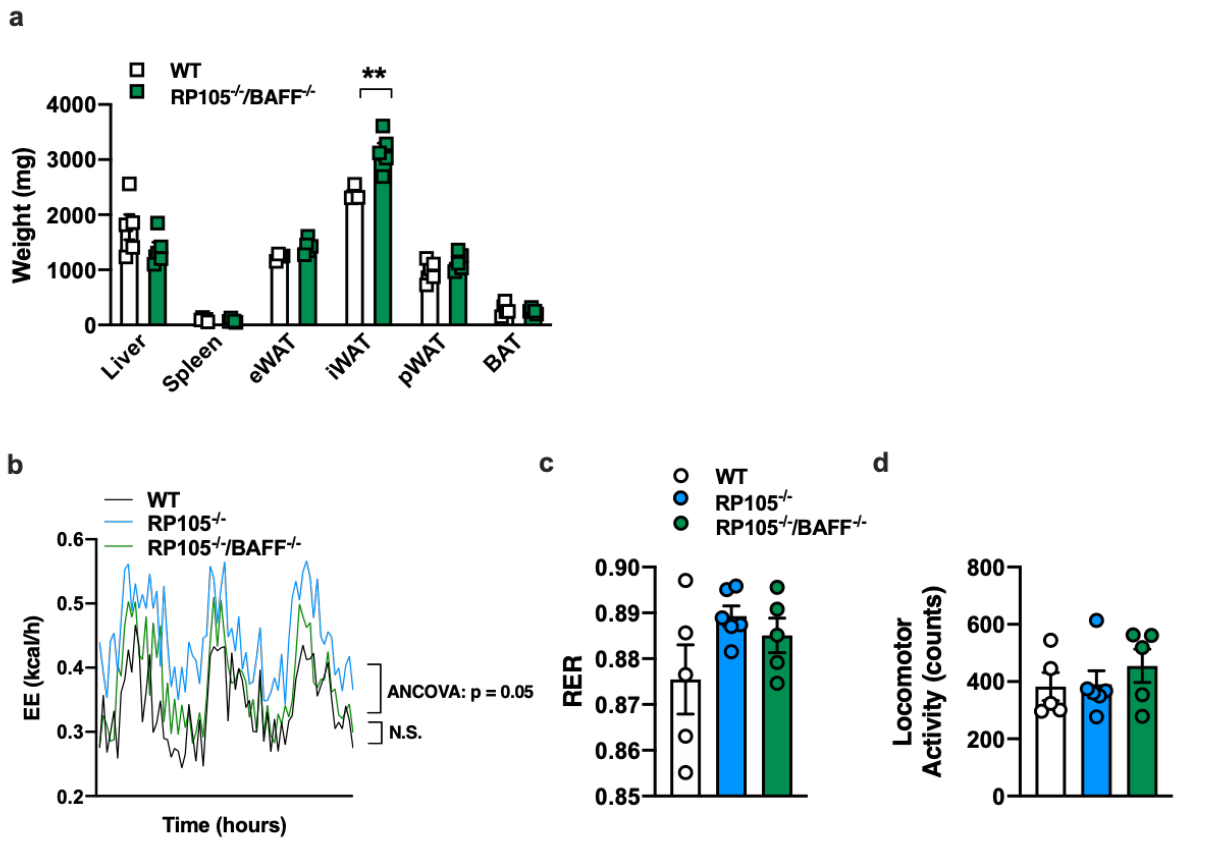

Supplementary Figure 3. Deletion of BAFF in RP105-deficient mice reverts energy expenditure. (**a**) RP105$^{-/-}$/BAFF$^{-/-}$ and WT mice were fed HFD for 16 weeks. Various tissue weights as indicated at time of harvest. (**b-d**) RP105$^{-/-}$, RP105$^{-/-}$/BAFF$^{-/-}$ and WT mice were fed a low-fat chow diet and analyzed in TSE Phenomaster systems for 3 days. (**b**) Cumulative energy expenditure. (**c**) RER. (**d**) Locomotor activity. (**a**) Representative of 3 independent experiments, n = 5/condition. (**b-d**) A single experiment, n = 5-6/condition. (**a-d**) For bar and line graphs, data represents mean +/- SEM. (**a, c-d**) Unpaired two-tailed Student's t-test. (**a**) ** *$p < 0.01$.* (**b**) Analysis of covariance (ANCOVA) with body weight as the covariate. *N.S. = not significant*. Source data are provided as a Source data file.

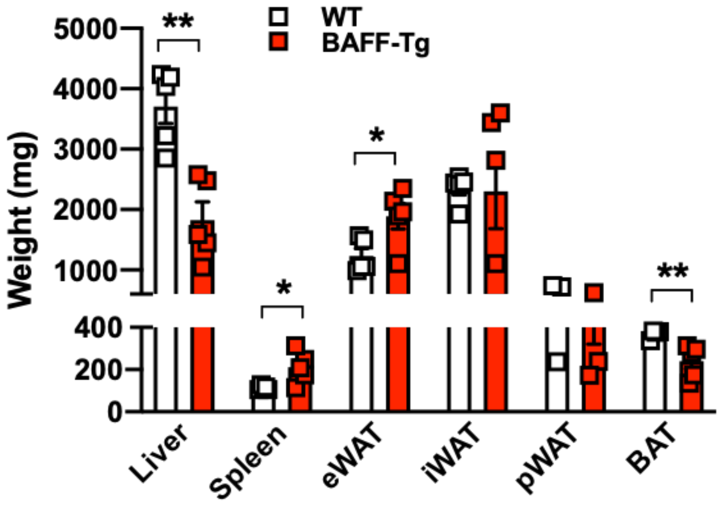

Supplementary Figure 4. Tissue distribution of HFD-fed BAFF-Tg mice. WT or BAFF-Tg mice were fed HFD for 20 weeks. Tissue weights as indicated at time of harvest. Representative of 3 independent experiments, n = 5/condition. For bar graph, data represents mean +/- SEM. Unpaired two-tailed Student's t-test. *$p < 0.05$, ** $p < 0.01$.* Source data are provided as a Source data file.

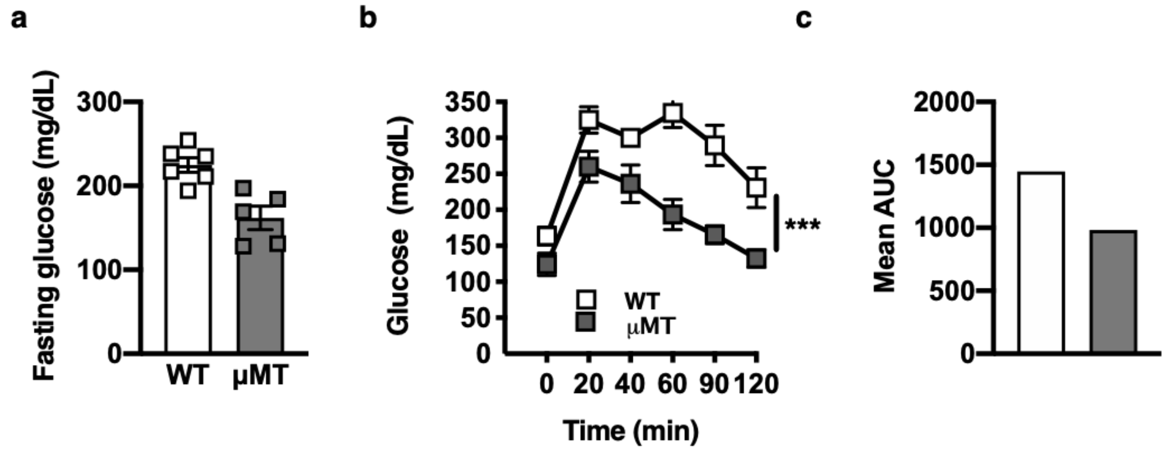

Supplementary Figure 5. μMT mice are protected from obesity-associated glucose dysmetabolism. WT and μMT mice were fed HFD for 20 weeks. (**a**) Fasting glucose at 20 weeks on HFD. (**b**) GTT at 12 weeks on HFD. (**c**) Combined mean AUC. (**a-c**) Representative of 4 independent experiments, n = 5-6/condition. (**a-b**) For bar and line graphs, data represents mean +/- SEM. (**a**) Unpaired two-tailed Student's t-test. *$p < 0.05$. (**b**) Area under the curve. *$p < 0.05$, **$p < 0.01$, ***$p < 0.001$. Source data are provided as a Source data file.

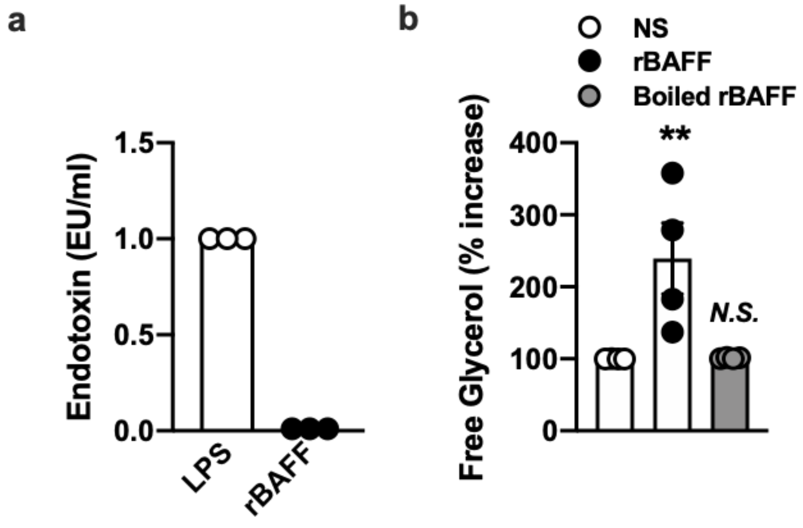

Supplementary Figure 6. Recombinant BAFF induction of subcutaneous white adipocyte lipolysis is independent of endotoxin contamination. (**a**) Endotoxin quantification of LPS (1 ng/ml), rBAFF (100 ng/ml) by chromogenic assay. (**b**) Quantified free glycerol by colorimetric assay in supernatants of primary subcutaneous white adipocytes treated with saline (NS), rBAFF (500 ng/ml) or boiled rBAFF (500 ng/ml, boiled for 30 min). (**a**) Representative of 2 independent studies, n = 3/condition (**b**) A single experiment, n = 4/condition. (**a-b**) For bar graphs data represents mean +/- SEM. (**a-b**) Unpaired two-tailed Student's t-test. (**b**) **$p < 0.01$. Source data are provided as a Source data file.

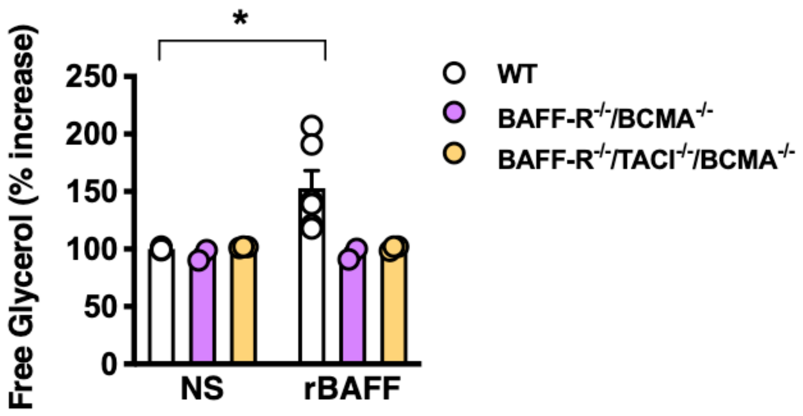

Supplementary Figure 7. BAFF/APRIL axis receptors modify BAFF sufficiency to induce subcutaneous adipocyte lipolysis. Quantified free glycerol by colorimetric assay in supernatants of primary subcutaneous white adipocytes from WT, BAFF-R$^{-/-}$/BCMA$^{-/-}$ or BAFF-R$^{-/-}$/TACI$^{-/-}$/BCMA$^{-/-}$ treated with saline (NS) or rBAFF (500 ng/ml), % increase relative to WT NS. Data combined from two independent experiments, n = 2-6/condition. For bar graphs data represents mean +/- SEM. Unpaired two-tailed Student's t-test. *$p < 0.05$. Source data are provided as a Source data file.

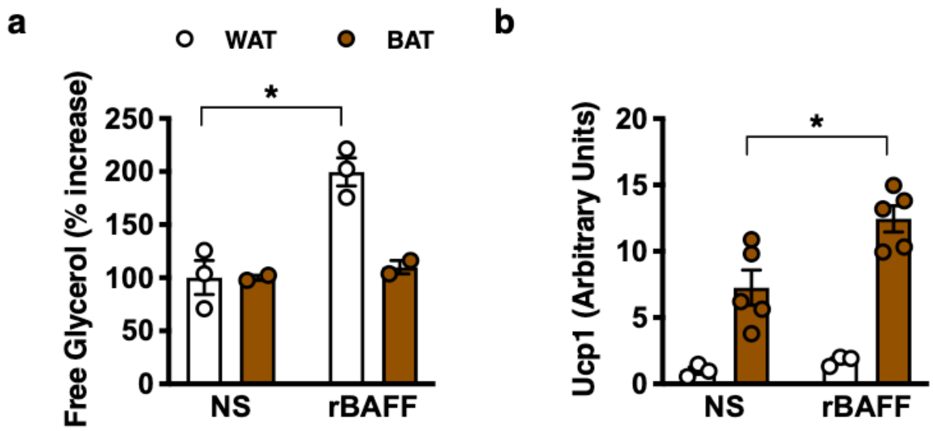

Supplementary Figure 8. BAFF's effects on lipolysis and Ucp1 induction are specific to subcutaneous white adipocytes and brown adipocytes respectively. Subcutaneous white adipocytes and brown adipocytes were treated with saline (NS) or rBAFF (500 ng/ml) for 24 hours and 6 hours respectively. (**a**) Free glycerol quantified by colorimetric assay, % increase relative to W. Adip NS. (**b**) *Ucp1* mRNA expression quantified by qPCR, data normalized to W. Adip *Ucp1* expression. (**a-b**) Representative of 2 independent experiments, n = 2-5/condition. (**a-b**) For bar graphs data represents mean +/- SEM. (**a-b**) Unpaired two-tailed Student's t-test. *$p < 0.05$. Source data are provided as a Source data file.

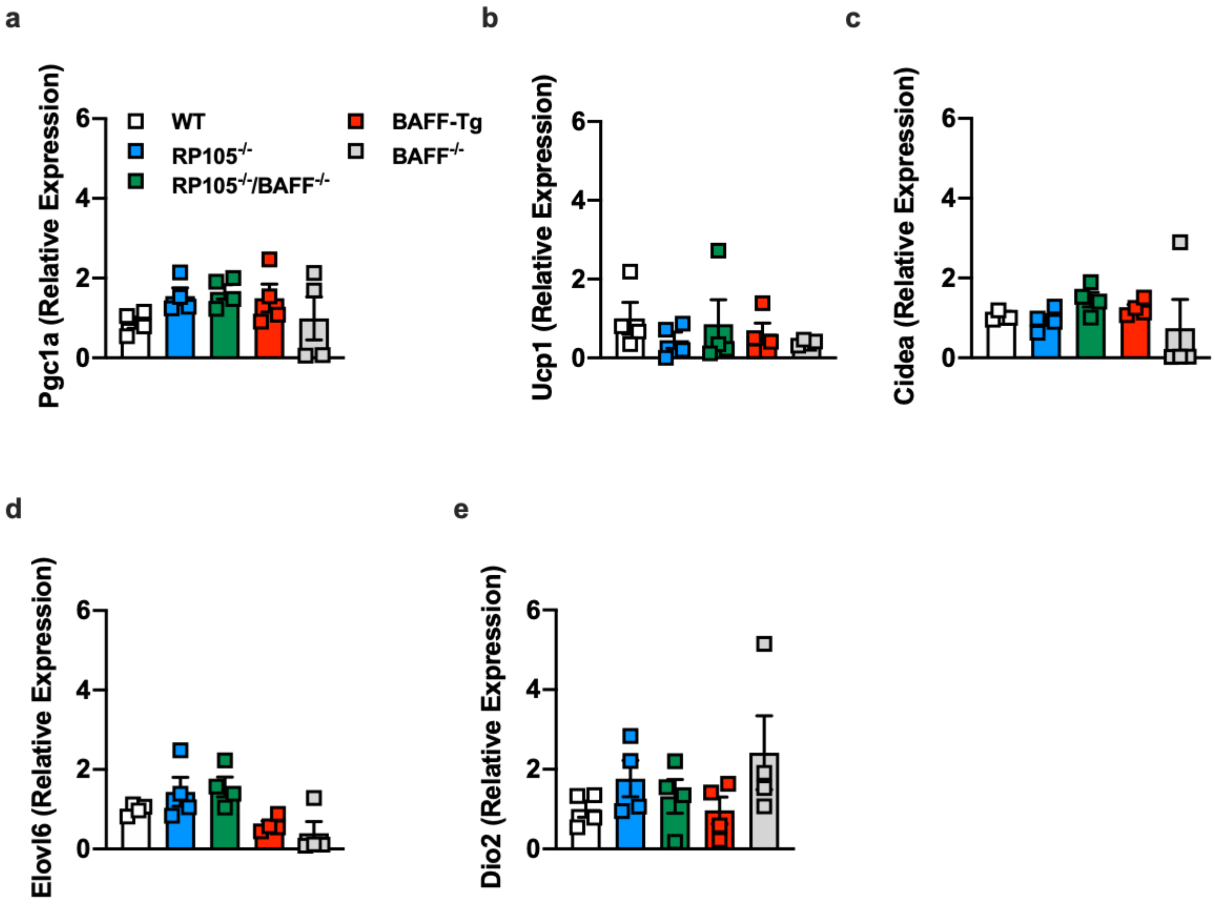

Supplementary Figure 9. Expression of browning/beiging-associated genes in subcutaneous WAT across various BAFF axis transgenic mouse models. (**a-e**) Differential mRNA expression, quantified by qPCR, of indicated genes in subcutaneous Wat from obese WT, RP105$^{-/-}$, RP105/BAFF$^{-/-}$, BAFF-TG, BAFF$^{-/-}$, APRIL$^{-/-}$, BAFF-R$^{-/-}$, TACI$^{-/-}$ and BCMA$^{-/-}$ mice as indicated. (**a**) Pgc1a mRNA expression, expression relative to WT. (**b**) Ucp1 mRNA expression, expression relative to WT. (**c**) Cidea mRNA expression, expression relative to WT. (**d**) Elovl6 mRNA expression, expression relative to WT. (**e**) Dio2 mRNA expression, expression relative to WT. (**a-e**) Representative of 3 independent experiments, n= 4/condition. (**a-e**) For bar graphs, data represents mean +/- SEM. (**a-e**) Unpaired two-tailed Student's t-test. Source data are provided as a Source data file.

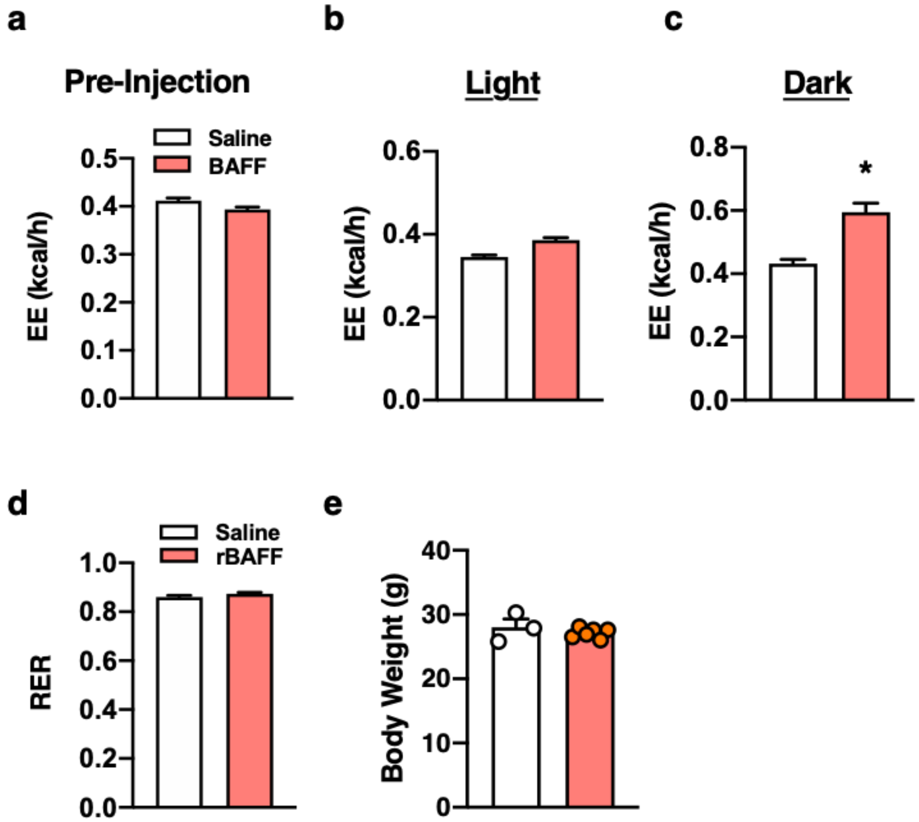

Supplementary Figure 10. Exogenous rBAFF administration augments energy expenditure. Lean CD-fed WT mice treated with rBAFF (2 μg/mouse) every other day for one week and monitored in TSE Phenomaster. (**a**) Combined energy expenditure prior to treatment. (**b**) Energy expenditure in light cycle. (**c**) Energy expenditure in dark cycle. (**d**) RER. (**e**) Body Weight one-week post-injections. (**a-e**) Representative of 2 independent experiments, n = 3-6/condition. (**a-e**) For bar graphs data represents mean +/- SEM. (**a-c**) analysis of covariance (ANCOVA) with body weight as covariate. *$p < 0.05$. (**d-e**) Unpaired two-tailed Student's t-test. Source data are provided as a Source data file.

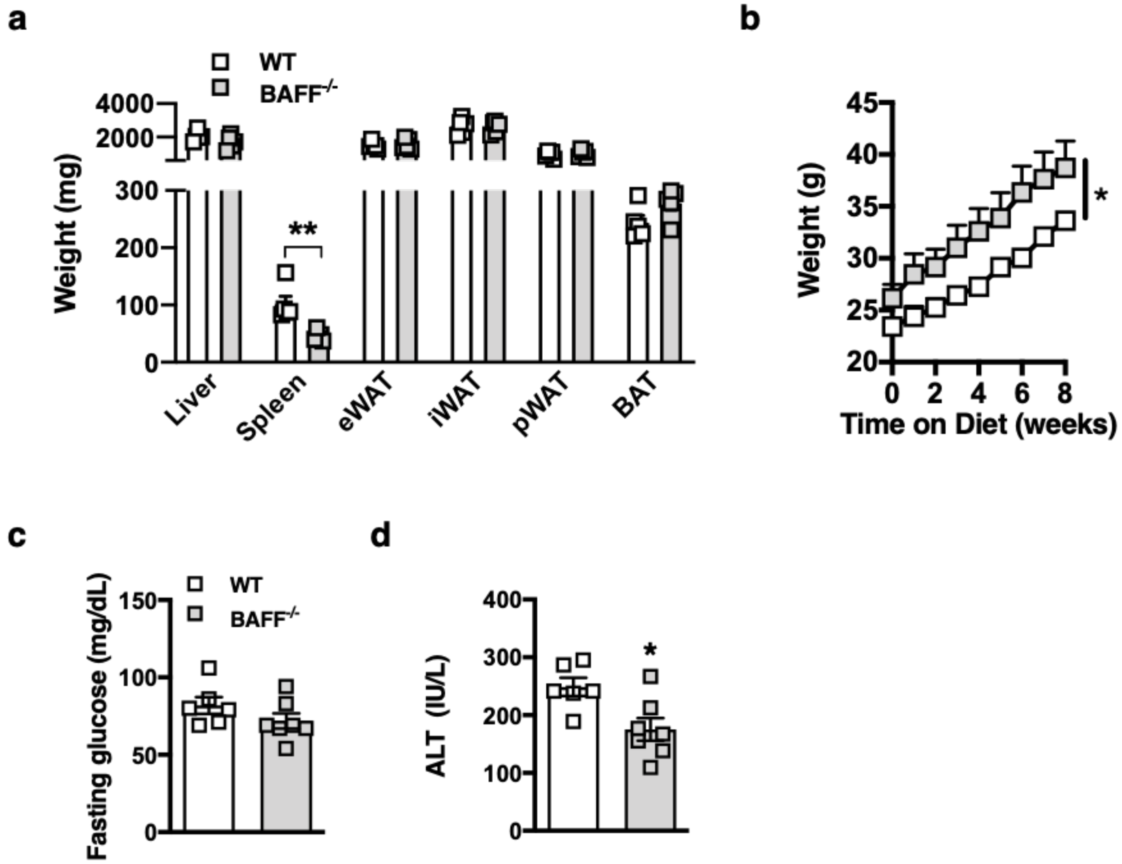

Supplementary Figure 11. BAFF-deficient mice exhibit increased body weight. (**a-d**) WT or BAFF$^{-/-}$ mice fed HFD for 20 weeks. (**a**) Tissue weight at time of harvest. (**b**) Body weight. (**c**) Fasting glucose at 13 weeks on HFD. (**d**) Systemic ALT at time of harvest. (**a-d**) Representative of 3 independent experiments, n = 5-6/condition. (**a, c-d**) For bar and (**b**) line graphs, data represents mean +/- SEM. (**a, c-d**) Unpaired two-tailed Student's t-test. *$p < 0.05$, ** $p < 0.01$. (**b**) Area under the curve, *$p < 0.05$. Source data are provided as a Source data file.

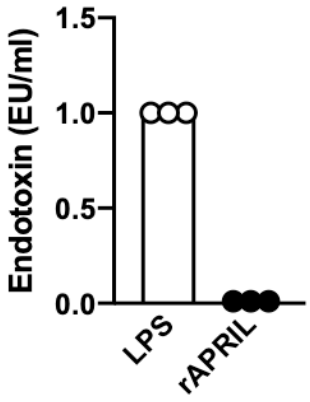

Supplementary Figure 12. Recombinant APRIL is devoid of detectable endotoxin contamination. Endotoxin quantification of LPS (1 ng/ml), recombinant APRIL (rAPRIL; 100 ng/ml) by chromogenic assay. Representative of 2 independent studies, n = 3/condition. For bar graph data represents mean +/- SEM. Source data are provided as a Source data file.

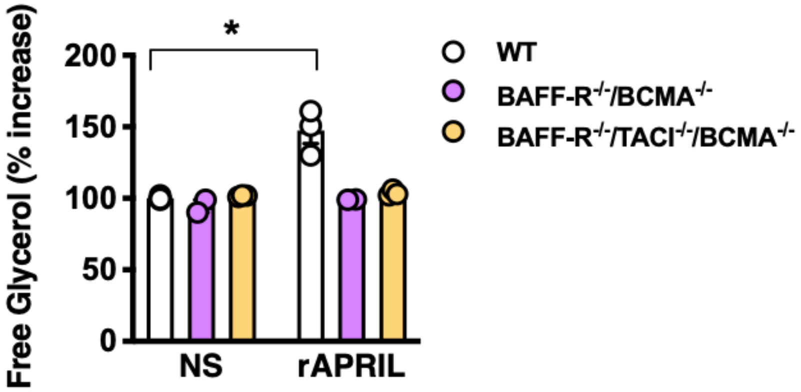

Supplementary Figure 13. BAFF/APRIL axis receptors modify APRIL sufficiency to induce subcutaneous adipocyte lipolysis. Quantified free glycerol by colorimetric assay in supernatants of primary subcutaneous white adipocytes from WT, BAFF-R$^{-/-}$/BCMA$^{-/-}$ or BAFF-R$^{-/-}$/TACI$^{-/-}$/BCMA$^{-/-}$ treated with saline (NS) or rAPRIL (500 ng/ml), % increase relative to WT NS. Data combined from two independent experiments, n = 2-6/condition. For bar graphs data represents mean +/- SEM. Unpaired two-tailed Student's t-test. *$p < 0.05$. Source data are provided as a Source data file.

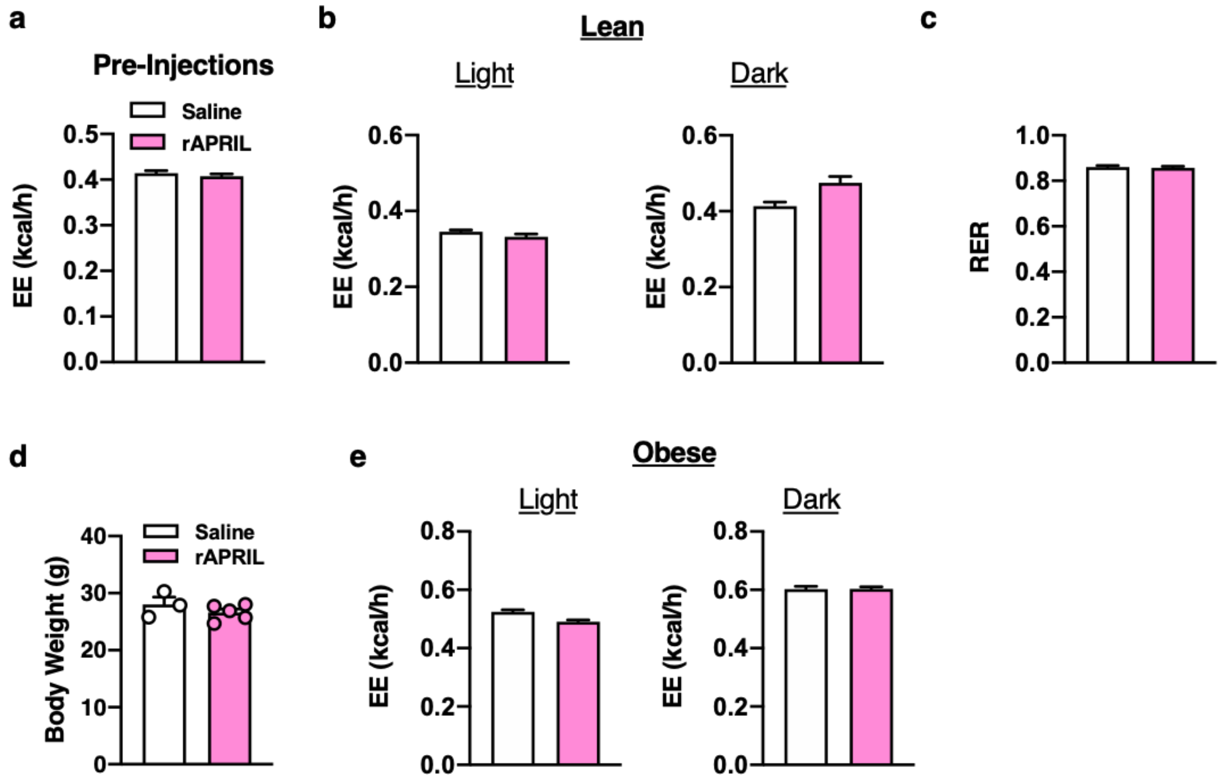

Supplementary Figure 14. Exogenous rAPRIL administration in lean mice augments energy expenditure. (**a-e**) Lean CD-fed WT mice were injected with rAPRIL (2 μg/mouse) every other day for one week and monitored in TSE Phenomaster. (**a**) Energy expenditure prior to treatment. (**b**) Energy expenditure in light and dark cycles. (**c**) RER. (**d**) Body Weight one-week post-injections. (**e**) Obese HFD-fed (20 weeks) WT mice were injected with rAPRIL (2 μg/mouse) every other day for one week and monitored in TSE Phenomaster. Energy expenditure in light and dark cycles. (**a-d**) Representative of 2 independent experiments, n = 3-5/condition. (**e**) A single experiment, n = 4-5/condition. (**a-e**) For bar graphs data represents mean +/- SEM. (**a-b, e**) Analysis of covariance (ANCOVA) with body weight as covariate. (**c-d**) Unpaired two-tailed Student's t-test. Source data are provided as a Source data file.

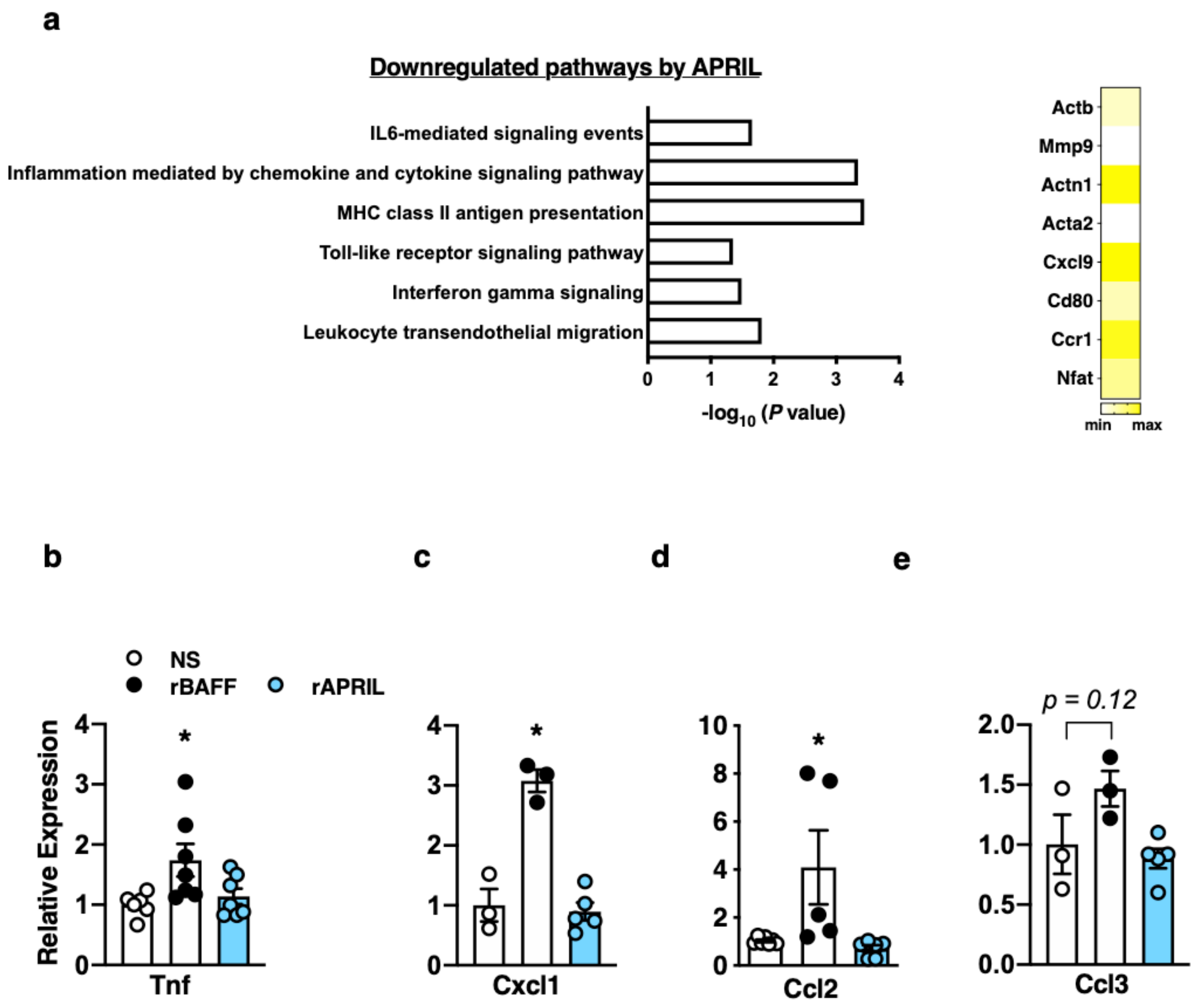

Supplementary Figure 15. APRIL does not induce inflammatory program in subcutaneous adipocytes. (**a**) Subcutaneous white adipocytes stimulated in the presence or absence of rAPRIL (500 ng/ml) for 24 hours and subjected to RNA-seq analysis. (**a**) APRIL-specific ontology pathways and heat maps of associated genes. (**b-e**) mRNA expression of indicated cytokines and chemokines quantified by qPCR, relative expression to NS. (**a**) A single experiment, n = 2/condition. (**b-e**) Representative of 3 independent experiments, n = 3-7/condition. (**b-e**) For bar graphs, data represents mean +/- SEM. (**b-e**) Unpaired two-tailed Student's t-test. *$p < 0.05$. Source data are provided as a Source data file.

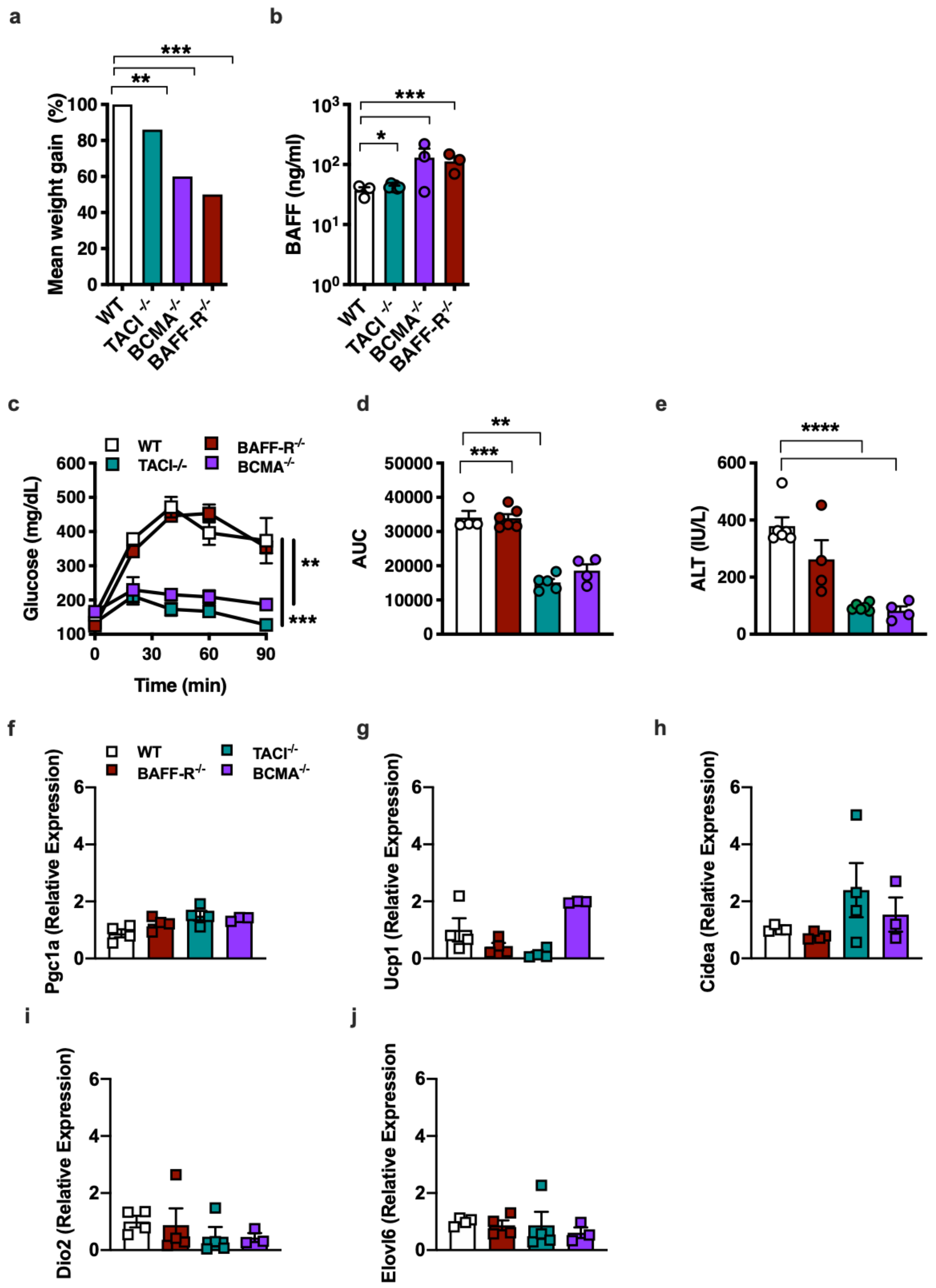

Supplementary Figure 16. BAFF-R$^{-/-}$, TACI$^{-/-}$, BCMA$^{-/-}$ mice are protected from HFD-driven weight gain. (**a-j**) BAFF-R$^{-/-}$, TACI$^{-/-}$, BCMA$^{-/-}$ and WT mice were placed on HFD for 24 weeks. (**a**) Mean weight gain. (**b**) Systemic BAFF levels quantified by ELISA in indicated lean mice. (**c**) GTT at Week 18 on a HFD. (**d**) AUC of GTT (**e**) ALT at time of harvest. (**f-j**) Differential mRNA expression, quantified by qPCR, of Ucp1-dependent thermogenic genes in subcutaneous WAT from obese WT, BAFF-R$^{-/-}$, TACI$^{-/-}$ or BCMA mice$^{-/-}$ as indicated. (**f**) Pgc1a mRNA expression, expression relative to WT. (**g**) Ucp1 mRNA expression, expression relative to WT. (**h**) Cidea mRNA expression, expression relative to WT. (**i**) Elovl6 mRNA expression, expression relative to WT. (**j**) Dio2 mRNA expression, expression

relative to WT. (**a-e**) Representative of 3 independent experiments, n = 3-6/condition. (**f-j**) Representative of 3 independent experiments, n = 3-4/condition. (**a-j**) For bar and line graphs data represents mean +/- SEM. (**a-b, d-e, f-j**) Unpaired two-tailed Student's t-test, (**c**) Area under the curve. (**a-e**) *$p < 0.05$, **$p < 0.01$, ***$p < 0.001$, ****$p < 0.0001$. Source data are provided as a Source data file.

	Time of surgery	1-year after bariatric surgery	P-value
**n**	30	22	
**Age (years mean +/- SEM)**	16.64 +/- 0.43		
**BMI (mean +/- SEM)**	50.15 +/- 2.25	38.69 +/- 2.21	***0.0007
**Glucose (mean +/- SEM)**	85.38 +/- 2.41	85.67 +/- 2.67	0.93
**HbA1c (mean +/- SEM)**	5.74 +/- 0.179		
**Aspartate aminotransferase (mg/dL mean +/- SEM)**	30.16 +/- 2.78	15.15 +/- 1.09	****<0.0001
**Alanine aminotransferase (mg/dL mean +/- SEM)**	35.51 +/- 5.32	24.77 +/- 3.59	0.122
**Total Cholesterol (mg/dL mean +/- SEM)**	161.5 +/- 5.33	155.5 +/- 6.68	0.45
**LDL (mg/dL mean +/- SEM)**	92.32 +/- 4.49	90.73 +/- 5.93	0.8
**HDL (mg/dL mean +/- SEM)**	31.29 +/- 1.09	47.08 +/- 1.87	****<0.0001
**Triglycerides (mg/dL mean +/- SEM)**	162.8 +/- 11.5	89.69 +/- 7.31	****<0.0001

Supplementary Table 1. Cohort characteristics of persons undergoing bariatric surgery. Data represents mean +/- SEM. Unpaired two-tailed Student's t-test.

	Lean	Persons with Severe Obesity	P-value
**n**	10	30	
**Age (years mean +/- SEM)**	19.40 +/- 0.78	16.64 +/- 0.43	**0.003
**BMI (mean +/- SEM)**	22.69 +/- 0.47	50.15 +/- 2.25	****<0.0001
**Glucose (mean +/- SEM)**	92.16 +/- 2.07	85.38 +/- 2.41	0.1968
**HbA1c (mean +/- SEM)**	4.85 +/- 0.07	5.74 +/- 0.179	**0.005
**Aspartate aminotransferase (mg/dL mean +/- SEM)**	23.81 +/- 2.17	30.16 +/- 2.78	0.28
**Alanine aminotransferase (mg/dL mean +/- SEM)**	16.09 +/- 2.84	35.51 +/- 5.32	0.08
**Total Cholesterol (mg/dL mean +/- SEM)**	141.6 +/- 5.71	161.5 +/- 5.33	****<0.0001
**LDL (mg/dL mean +/- SEM)**	68.50 +/- 4.43	92.32 +/- 4.49	***0.0003
**HDL (mg/dL mean +/- SEM)**	54.75 +/- 2.25	31.29 +/- 1.09	****<0.0001
**Triglycerides (mg/dL mean +/- SEM)**	73.38 +/- 6.68	162.8 +/- 11.5	0.38

Supplementary Table 2. Cohort characteristics of lean and severely obese persons. Data represents mean +/- SEM. Unpaired two-tailed Student's t-test
